# Crop diversity and susceptibility of crop fields to elephant raids in eastern Okavango Panhandle, northern Botswana

**DOI:** 10.1002/ece3.9910

**Published:** 2023-03-21

**Authors:** Tiroyaone A. Matsika, Gaseitsiwe S. Masunga, Anastacia Makati, Graham McCulloch, Amanda Stronza, Anna C. Songhurst, Joseph A. Adjetey, Motshwari Obopile

**Affiliations:** ^1^ Botswana University of Agriculture and Natural Resources Gaborone Botswana; ^2^ Ecoexist Trust Maun Botswana; ^3^ Okavango Research Institute, University of Botswana Maun Botswana; ^4^ Texas A & M University College Station Texas USA; ^5^ University of Oxford Oxford UK

**Keywords:** crop raiding, crop species, field risk value, food security, human–elephant conflict, incidence risks

## Abstract

Elephants frequently raid crops within their ranges in Africa and Asia. These raids can greatly impact agricultural productivity and food security for farmers. Therefore, there is a need to explore cost‐effective measures that would reduce the susceptibility of crops and agricultural fields to elephant raiding, and further promote sustainable human–elephant coexistence. Previous studies have examined the susceptibility of crop fields to elephant raids using field characteristics such as field size and proximity to water sources. However, there are limited studies investigating how different crop types, individually and in their combinations, influence crop susceptibility to elephant raiding. This study utilized data collected from crop fields raided by the African savanna elephant (*Loxodonta africana*) between 2008 and 2018 in the eastern Okavango Panhandle, northern Botswana. Data on crops grown, number of crop‐raiding incidences for each crop, and elephant raiding incidences were recorded for each field assessed. Incidence risks (IR) and field risk value (RV) were computed using an adaptive epidemiological approach. The results showed that elephant raiding incidents varied significantly amongst crop types over space and time (*p* < .0001). Cereal crops (millet: *Eleusine conaracana*, maize: *Zea mays*) incurred a higher number of crop‐raiding incidents compared with leguminous crops (cowpea: *Vigna unguiculata*; groundnut: *Arachis hypogea*). Field RVs significantly varied depending on which crop was present in the field. There was a significant negative correlation between the number of crop types and the susceptibility of the field to raiding (*r* = −0.680, *p* < .0001). Our results suggest that the susceptibility of the fields to elephant raids could be minimized by selecting crop types and combinations less susceptible to elephant damage, thus enhancing food security for local subsistence farmers.

## Graphical abstract

Susceptibility of the fields to elephant raids could be minimized by selecting crop types and combinations less susceptible to elephant damage. Therefore, mitigations benefiting both farmers and elephants form an essential component of coexistence and significantly influence the success of many conservation projects and enhancing food security for subsistence farmers.

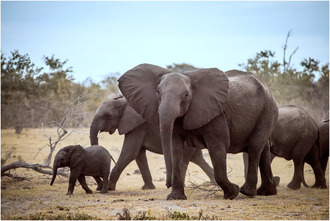

## 1 INTRODUCTION

Human–elephant conflict (HEC) is a major concern in areas where elephant and human‐inhabited ranges overlap. Competition for space and resources is the main underlying driver of HEC (Songhurst & Coulson, 2014), with an array of social, ecological, economic, and political factors also contributing (Songhurst, 2017). The negative interactions arising from such conflicts include direct and indirect impacts, such as crop losses (Bond, 2015) and consequent potential income from the lost crop (Gontse et al., 2018). When the cost of coexisting with elephants far outweighs the benefits (Mayberry et al., 2017), crop loss can lead to resentment towards elephants by local communities (Kansky & Knight, 2014).

Botswana has the largest population of African savanna elephants (*Loxodonta africana*) in the world, with current estimates between 130 and 150 thousand individuals (Chase et al., 2016; Thouless et al., 2016) ranging throughout protected and unprotected areas. In much of the elephant range outside of protected areas, people are living and farming, with the main livelihood being subsistence farming (Gontse et al., 2018). In the eastern Okavango Panhandle in northern Botswana, the location of this study, HEC incidents are frequent, with crop raiding being the most common form of HEC (Songhurst et al., 2016).

Many studies have identified and elaborated on factors influencing the susceptibility of crops and agricultural fields to crop raiding by wildlife (Jackson et al., 2008; Naughton et al., 1998; Sitati et al., 2005; Songhurst & Coulson, 2014). Different methods have been used to assess and characterize such susceptibility to elephant crop raiding. These methods included comparative assessments of raided and nonraided fields (Mosojane, 2004), determination of the influence of spatiotemporal characteristics, and the effect of field size, location, and mitigation measures on field and crop susceptibility (Buchholtz et al., 2019; Jackson et al., 2008; Sitati et al., 2005; Songhurst & Coulson, 2014). Studies found out that large fields with fences were more susceptible to crop raiding than small fields and that consistent guarding, use of chili, fire, and noise were more effective than wire fences in protecting crops against elephant raids (Montgomery et al., 2022; Sitati et al., 2005). In a study carried out in the eastern Okavango Panhandle, the distance of a field to a main elephant movement path and the age of the field were found to be the main factors influencing crop raiding (Songhurst & Coulson, 2014). In Hwange region in Zimbabwe, the distance from the refuge or protected area was found to be a driver of crop raiding by elephants (Guerbois et al., 2012). Fewer studies, however, have determined the influence of crop types and cropping strategies on patterns of elephant crop raiding. In Uganda and Kenya, it was found that crop types can be good predictors of frequency of elephant raids on crop fields (Naughton et al., 1998; Sitati et al., 2005), as some crop types such as cereal can be more attractive to elephants than other crop types. Some crop types tend to be repulsive to elephants because of the high concentration of secondary metabolites or chemical defenses they possess in their tissues (Gross et al., 2016; Owen‐Smith & Chafota, 2012). Other factors that can be influential in driving patterns of elephant crop raids include season, the size of the farm, and proximity to protected areas (Monney et al., 2010; Tiller et al., 2021).

Priston and Underdown (2009) and Nijman and Nekaris (2010) tried an epidemiological predictive model to predict the pattern and probabilities of animal crop raids based on individual crops and the impact of those crops when planted together in a farm. Priston and Underdown (2009) disputed the fact that crop raiding was crop species‐dependent and that it had differential rates of occurrences. Nijman and Nekaris (2010) improved on the accuracy of the Priston and Underdown's (2009) method. This improvement presents an opportunity to use the method and investigate the susceptibility of crop fields to elephant raiding. The epidemiological approach method uses the data derived from the actual elephant raiding events to identify which crop is more vulnerable (incidence risk) and how a combination of crop types collectively influences the vulnerability of the whole field or farm (RV). The accuracy of the determination is improved by computing incidence risks (IRs) from a larger sample size or the number of farms rather than relying on fewer sample sizes or secondary experience from the farmer (Regmi et al., 2013). Even though the epidemiological approach has been used largely on larger primates as crop‐raiding wildlife, the method has also proved to be very precise for other crop raiding animals (Nijman & Nekaris, 2010).

Currently, many farmers depend on active guarding of crops to deter elephants, using a variety of mitigation measures, but these can be labour‐intensive and resource‐consuming (Bond, 2015). Such mitigation measures include the use of bees (King et al., 2016) and processed chili (Osborn, 2002; Parker & Osborn, 2006; Chang et al., 2016; Pozo et al., 2017). Elephants are also averse to mature chili crops, which can add to the deterrent toolbox, and be planted as a buffer to other plants that it is intercropped with (Matsika et al., 2020). The role of different crop combinations and crop choice by the farmer in relation to reducing the susceptibility of a field to elephant raiding is currently poorly understood.

The aim of this study was to investigate (a) the susceptibility of individual crops to elephant crop raiding; (b) the influence of crop diversity on field's susceptibility to elephant raiding; and (c) spatiotemporal patterns of elephant crop raiding in the eastern Okavango Panhandle. Through this investigation, we intended to answer the questions (i) how susceptible are individual crops to crop raiding? (ii) how crop diversification influences the vulnerability of a farmer's field to crop raiding and (iii) how elephant crop‐raiding incidents are distributed over time and space in the eastern Okavango Panhandle? On the basis of the three questions above, the study predicted that: (1) the level of susceptibility of individual crops to elephant crop raiding would differ significantly between the crop types, with cereal crops more susceptible to crop raiding than other crops; (2) high crop diversity, especially of functional groups, would be associated with low susceptibility of crop fields to elephant raiding; (3) elephant crop raiding incidents would show a decline over years as farmers increase diversity of crop types in their fields, and that villages whose fields are located within or near prominent elephant corridors or paths would experience higher incidents of crop raiding than fields located elsewhere.

## 2 MATERIALS AND METHODS

### 2.1 Study area

**FIGURE 1:**
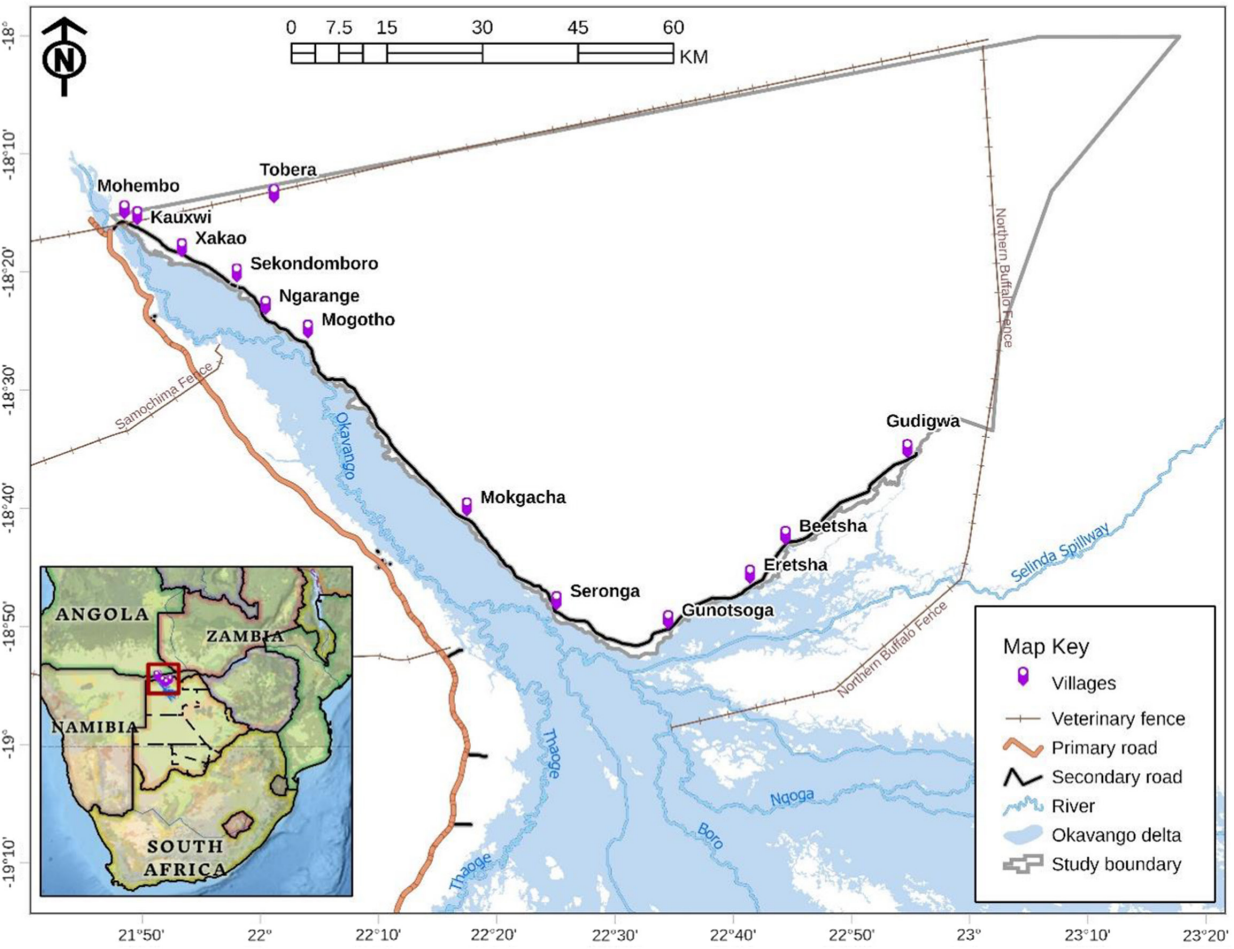
Eastern Okavango Panhandle, showing the 13 villages where field data were collected. (Source of basemaps: Esri, USGS, FAO; 2021).

The study took place in the eastern Okavango Panhandle, in northern Botswana (Figure 1). It comprises three wildlife management areas (WMAs), namely NG11, NG12, and NG13, and covers an area of around 8500 km^2^. These WMAs are not fenced, and human settlements, agricultural, and tourism activities are allowed. The nearby fully protected area in the form of a national park is Moremi Game Reserve, which is situated far south. The large distance between this reserve and the study area renders its effect as a source of elephants to the study area negligible. There are 14 villages spread over a distance of 162 km from Mohembo‐East in the far north of the Panhandle to Gudigwa in the southeast, including Mohembo‐East, Xakao, Kauxwi, Kaputura, Sekondomboro, Ngarange, Tobera, Mogotho, Mokgacha, Seronga, Gunotsoga, Eretsha, Beetsha, and Gudigwa. The villages and settlements form a linear pattern along the perennial Okavango River and its tributaries. The fenced international boundary between Namibia and Botswana borders these villages in the north, Northern Buffalo veterinary cordon fence in the east and the Okavango River in the west and south. These physical boundaries enclose the villages and restrict the movement and dispersal of elephants as they come and leave the Okavango River, which provides them with permanent surface water to drink.

The Okavango Delta receives an annual rainfall of about 450 mm. The annual mean minimum and maximum monthly temperatures are 25°C and 35°C, respectively (Department of Meteorological Services, Botswana, 2016).

The estimated population of elephants in the area is 18,000 (Songhurst, 2017), and the estimated population of people is 16,000 (Central Statistics Office, Botswana, 2011). The area comprises diverse ethnic groups, mainly Hambukushu, Wayei, and Basarwa. These people primarily depend on subsistence agriculture and fishing for sustaining their livelihoods (Motsholapheko et al., 2012). The main livelihood in the area is subsistence agriculture. Farmers plant a variety of crops, mainly maize (*Zea mays*), sorghum (*Sorghum bicolor*), millet (*Eleusine conaracana*), watermelon (*Citrullus lanatus*), pumpkin (*Cucurbita spp*), groundnut (*Arachis hypogea* L), and cowpea (*Vigna unguiculata*), with maize, millet, and sorghum being the primary crops planted (Marumo et al., 2014). The study area is a hotspot of human–elephant conflict because of the intense competition for space and food resources; the conflict is characterized by high incidents of crop raiding by elephants, property damage and injury or death of people, and injury or death of elephants (Buchholtz et al., 2019; Songhurst et al., 2016).

### 2.2 Data collection

Data on elephant crop raiding were collected by A. Songhurst and the Ecoexist team between 2008 to 2018, in 1347 crop fields distributed across 14 villages in the eastern Okavango Panhandle. All crop fields selected for this study were fenced with a traditional bush fence, which is a physical wall around a field erected from branches cut off from bushy trees and shrubs in the area. The fields were not using other elephant crop‐raiding deterrents apart from the bush fence and crops that were planted.

Field assessments were performed during the late wet seasons when most crops in the crop fields have attained maturity and harvestable stage, a stage when elephant damage on crops is at its peak (Snyder et al., 2021). Most crop raids by elephants in Eastern Okavango Panhandle occur in the late wet season when crops have reached maturity and resources (forage and surface water) in wildlife areas are diminishing because of the onset of the dry and cold season.

Data collection followed the IUCN data collection protocol designed by Hoare (1999). Following the Protocol's Method No. 2, the actual assessment of crop raiding by elephants was measured and quantified by the investigators instead of total reliance on a verbal report from the farmers. Weekly routine checks made by the enumerators ensured that all damages were recorded even if they were not reported and attended to immediately. Because farmers were informed about the study and trained on the importance of the data, issues of not reporting to the enumerators and investigators were few. This approach allowed for the quantification of the actual losses and reduced bias. Around the crops' maturity stage before harvest, field assessment surveys were made where elephants were reported to have invaded fields. Data on crops grown, number of crop‐raiding incidents for each crop, and damage details were recorded for each field assessed. Crops were classified as available (code = 1) or unavailable (code = 0) in the farm prior to the raid incident. Crops were also recorded as damaged (code = 1) or not damaged (code = 0). This allowed us to determine the total number of crops a farmer planted and which amongst the available crops were evidently damaged.

Crops were categorized into three groups for analysis, which were cereals (sorghum, maize, and millet), melons (watermelon and pumpkin), and legumes (groundnut and cowpea).

### 2.3 Data analysis techniques

Adaptive epidemiological models (Nijman & Nekaris, 2010; Priston & Underdown, 2009) were used to determine each crop's susceptibility to elephant raiding and the susceptibility of the farmers' fields to elephant raiding. These models are reliable in predicting the likelihood of crop raiding and vulnerability of fields to crop raiding depending on different types of crops grown within the fields (Nijman & Nekaris, 2010). R version 3.5.1 + R studio statistical package (R Core Team, 2013) was used to map yearly raiding incidents for villages.

Data used in the adaptative epidemiological models were from crop fields that were accessed by elephants and reflected whether damage occurred on individual crops or not. Crops were coded as planted and available (1) or not planted or absent (0). The incidence risk (IR) of crop raiding is defined as the ratio of new occurrences over time to the crops at risk over that period (Nijman & Nekaris, 2010). The IR was thus calculated using the following formula:

$$\mathrm{IR}=\frac{\mathrm{new}\;\text{occurence over}\;a\;\text{period of time}\left(a\right)}{\text{crop}\;\mathrm{at}\;\text{risk of being raided}\;\mathrm{but}\;\text{was not raided}\left(b\right)}$$

where

a—number of fields in which elephants damaged the crop.

b—number of fields where the crop was present and available for potential crop raiding.

The highest IR that a crop could have is 1, which indicates that a crop is highly susceptible to crop raiding, whereas the lowest IR of 0, indicates low susceptibility to crop raiding.

Risk value (RV) for each field was then calculated by summing up of IRs for all the crops present in the field (Nijman & Nekaris, 2010). A higher RV indicated higher susceptibility of a field to elephant raiding. Pearson's correlation was used to establish the strength and direction of relationship between independent variables (presence of a particular crop) and dependent variables (crop raiding) at *p* < .05 (Hauke & Kossowski, 2011).

$$\mathrm{RV}=\sum \;{\mathrm{IR}}_{\left(\mathrm{all}\;\text{crops present in the field}\right)}$$

The correlation coefficient was computed as:

$$r=1-\frac{6\sum d^{2}}{n\left(n^{2}-1\right)}$$

where:

*d*—the difference between the two ranks of each observation.

*n*—the number of observations (Hauke & Kossowski, 2011).

The outcome of correlation analysis ranges from −1 to +1. A coefficient closer to +1 means a strong positive relationship between the tested variables. On the contrary, a coefficient closer to −1 means a strong negative relationship (Hauke & Kossowski, 2011). The data on the incidence of elephant raiding (dependent variable) were analyzed using the Generalized Linear Models (GLM) procedure in SPSS (Version 23.0.0) to test for variation on the following parameters: crop; year; and crop × year interactions (independent variables). Villages were considered to be a random effect in our analysis. Differences in the incidence of raiding between crops (averaged across the year) and between years (averaged across crops) were significant if their respective 95% confidence intervals (CI) did not overlap. Mann–Whitney U test was used to compare risks of raiding (IR) for the crops.

## 3 RESULTS

### 3.1 Farmer cropping choice

**FIGURE 2:**
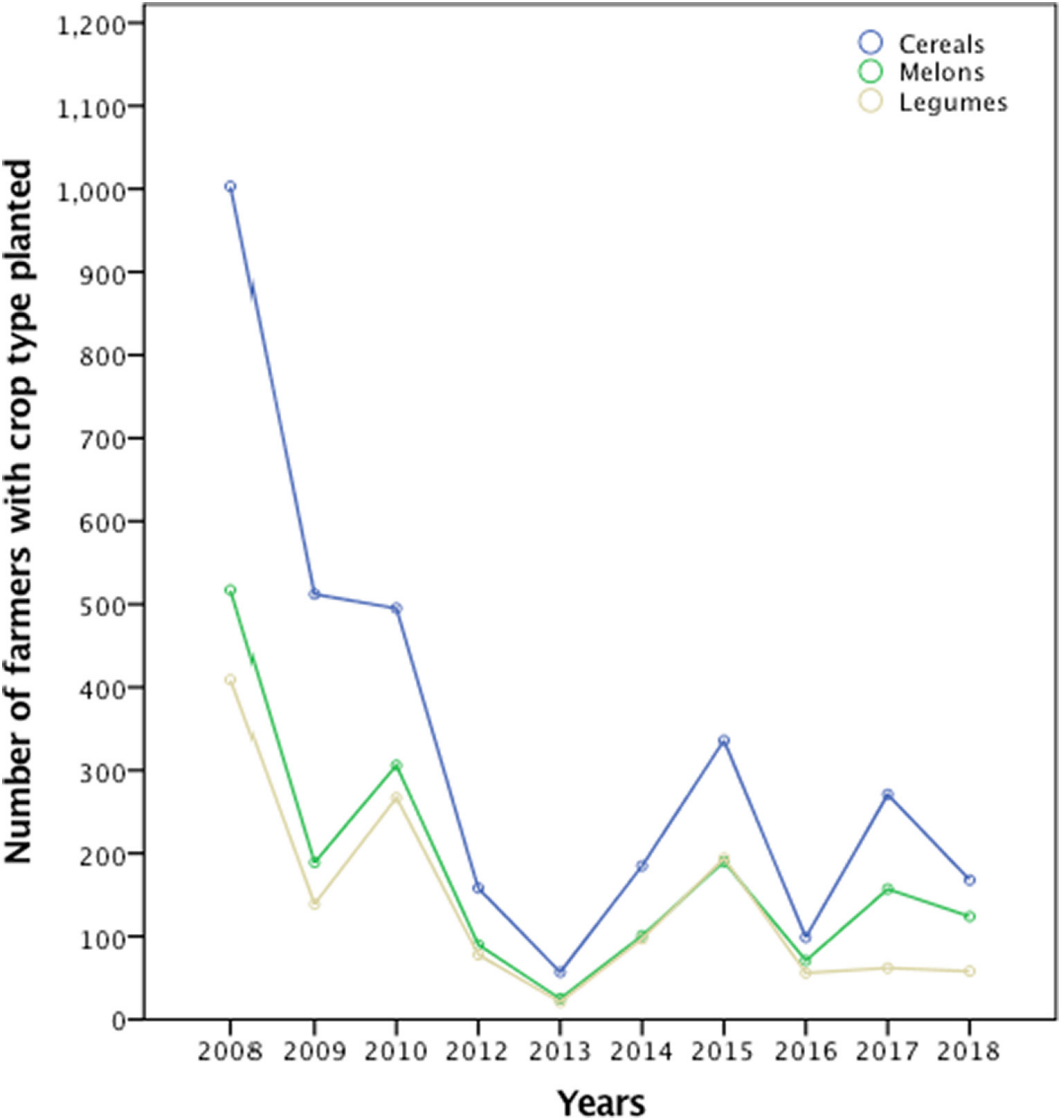
Number of farmers who planted from different crop families between 2008 and 2018.

Cereals [millet (*Eleusine conaracana* L. *Gaertn*) (94.51%), maize (*Zea mays* L.) (81.29%), grain sorghum (*Sorghum bicolor* L.) (67.78%), and sweet sorghum (*Sorghum bicolor*) (53.08%) combined] were the crop types most preferred by elephant, followed by cucurbits [watermelon (*Citrullus lanatus* sp) (78.10%) and pumpkin (*Cucurbita* spp.) (53.16%) combined], and then legumes [groundnut (*Arachis hypogea* L.) (38.68%) and cowpea (*Vigna unguiculata* (L.) Walp.) (63.85%) combined]. There has been a dramatic decline in the number of farmers that planted different crop types between 2008 and 2018. The number of farmers who planted cereals also declined from over 1000 farmers in 2008 to <300 in 2018. The number of farmers that planted melons also declined from over 500 to <200, as well as for legumes, which declined from 400 to <100 farmers within the study period (Figure 2).

### 3.2 Incidence of elephant raiding over time

**FIGURE 3:**
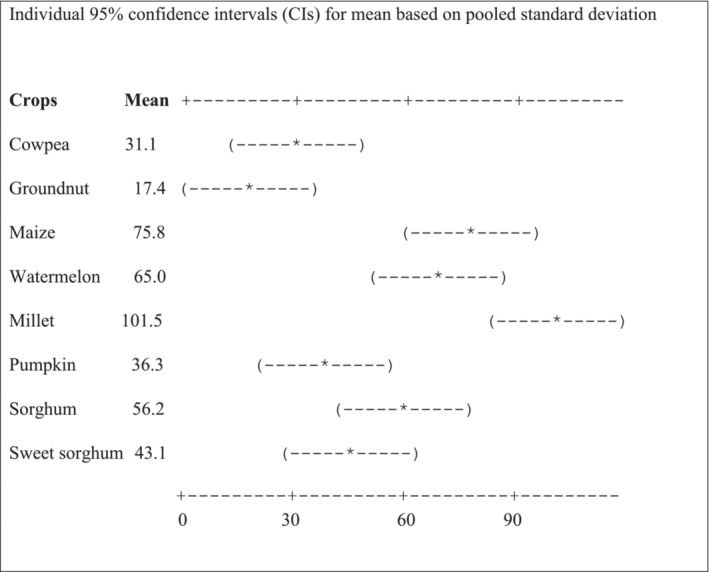
Mean of crop‐raiding incidences on different crops averaged across years of sampling (2008–2018).

There was a significant difference in the number of crop‐raiding incidents between crop types (*F* = 9.16, *df* = 7, *p* < .0001) and year (*F* = 19.23, *df* = 9, *p* < .0001, Appendix S1–S5). A significantly higher number of elephant crop‐raiding incidents occurred on millet followed by maize, watermelon, and sorghum compared with cowpea and groundnut as indicated by nonoverlapping confidence intervals (Figure 3). The lower number of incidents were observed on groundnut and cowpea, respectively. The frequency of cultivation of a particular crop did not correlate with the field's susceptibility to elephant raiding (*r* = 0.017, *n* = 792, *p* = .63; Appendix S1–S5).

**FIGURE 4:**
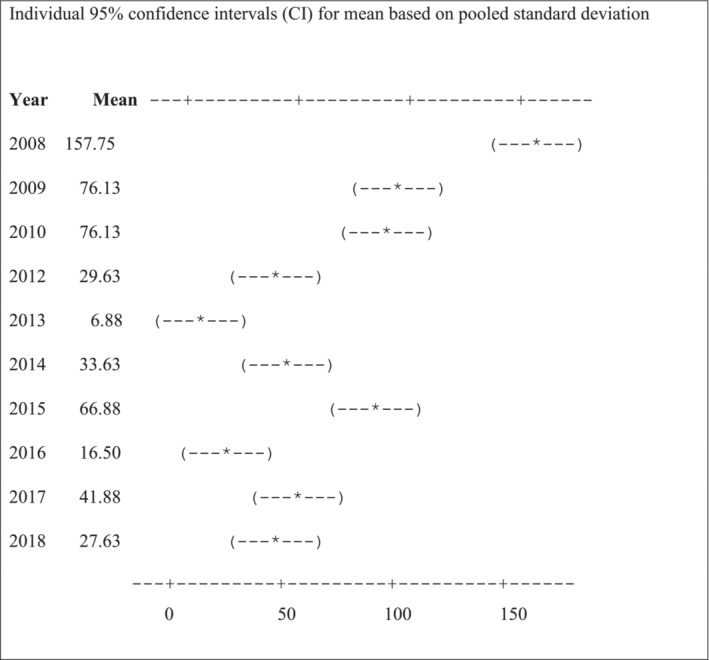
Mean of elephant crop‐raiding incidences recorded on farmers' fields between 2008 and 2018 in the eastern Okavango Panhandle.

A higher number of incidences of crop raiding by elephants occurred in 2008, and then in 2009 and 2010, and 2015 (Figure 4). A significantly lower number of incidents occurred in 2013 and 2016.

**FIGURE 5:**
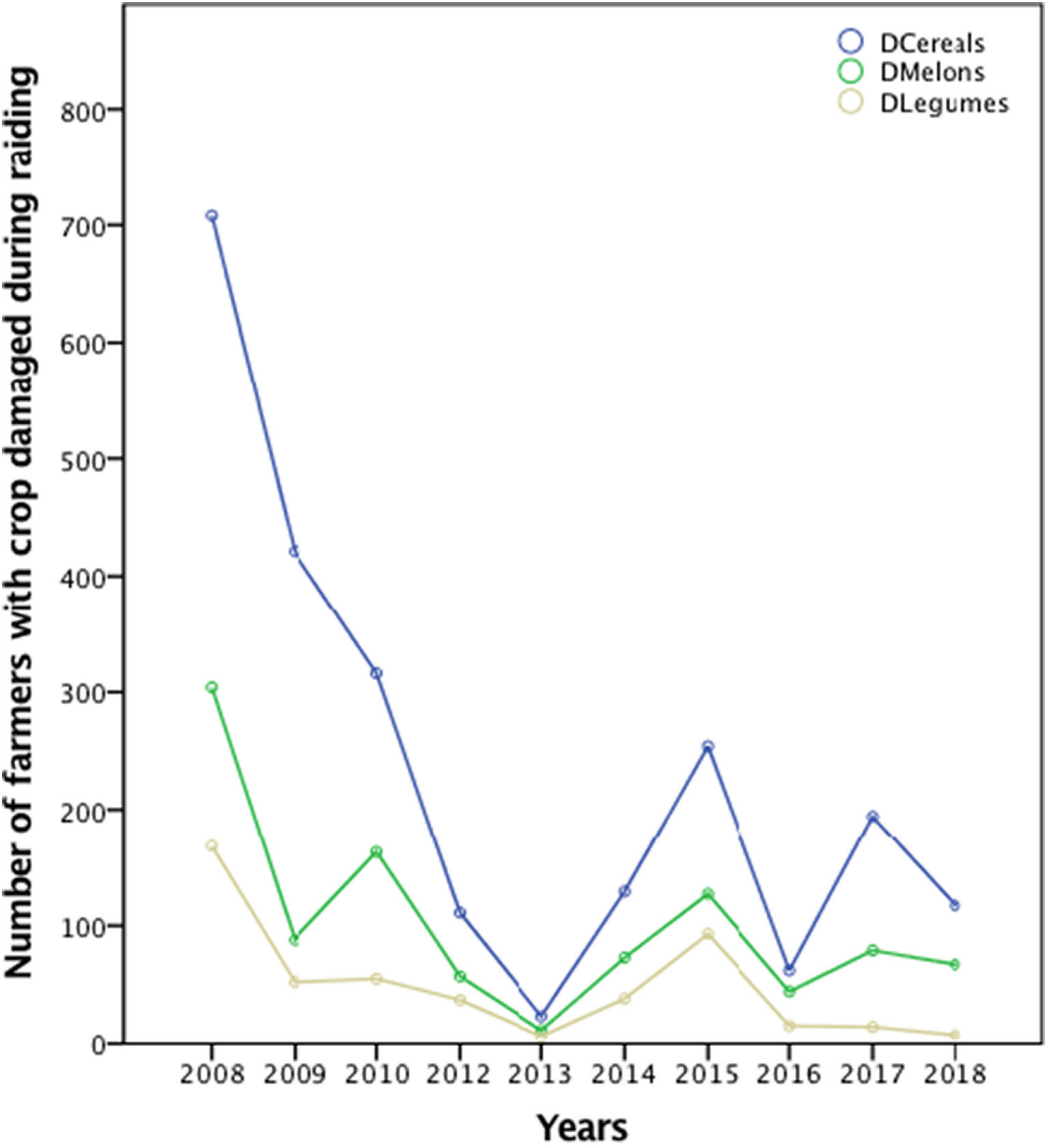
Number of farmers whose crops were destroyed between 2008 and 2018.

Cereals (millet, maize, sorghum, and sweet sorghum) were consistently the most raided crop types followed by melons (watermelon and pumpkin) then legumes (groundnut and cowpea) (Figure 5). Generally, there was a consistent decline in the number of farmers whose crops were destroyed by elephants between 2008 and 2018, also indicating a decline in the number of crop‐raiding incidents. The number of farmers whose cereal crops were destroyed decreased from 700 to 200; for melons, the numbers decreased from 300 to 100, and for legumes, the numbers decreased from 200 to <10 between 200 and 2018.

### 3.3 Assessing susceptibility of crops to elephant raiding

The Incident Risk (IR) was calculated for the eight crop types or species (Table 1). Millet was the most susceptible crop to elephant raiding because of higher IR and was followed by maize, sorghum, watermelon, sweet sorghum, and pumpkin, respectively. Cowpea and groundnut were the least susceptible crops to elephant raiding. The risk of raiding for a crop significantly and positively correlated with the actual raid incidents for that particular crop (*r* = 0.39, *n* = 792, *p* < .0001; Appendix S1–S5). As mentioned above, the availability of a crop in the field did not influence the field's susceptibility to elephant raiding.

**TABLE 1:** Susceptibility of individual crops to elephant raiding measured by incidence risk (IR) between 2008 and 2018 (*n* = 1347).

Crop	Number of farms with crop available (b)	Percentage of farms with crop	Number of farms where crop was raided (a)	Risk of raiding for crop (IR)
Millet	1273	94.51	1015	0.80
Maize	1095	81.29	757	0.69
Sorghum	913	67.78	562	0.62
Watermelon	1052	78.10	649	0.62
Pumpkin	716	53.16	362	0.51
Sweet sorghum	715	53.08	431	0.60
Cowpeas	860	63.85	311	0.36
Groundnut	521	38.68	174	0.33

Using Mann–Whitney U test, the above scores for risk of raiding for crops were compared against millet, which was the most susceptible crop (Table 2). There was a significant difference in the risk of raiding for all crops in relation to millet.

**TABLE 2:** Mann–Whitney U test comparison on risk of raiding for millet against other crops.

IR comparisons	*p*‐value
Maize vs. millet	.036
Groundnuts vs. millet	<.0001
Watermelon vs. millet	.001
Cowpea vs. millet	<.0001
Pumpkin vs. millet	<.0001
Sweet sorghum vs. millet	<.0001
Sorghum vs. millet	<.0001

*Note*: Crop comparisons computed at medians of Maize—.5, Groundnut—.05, Watermelon—.4, Cowpea—.11, Pumpkin—.03, Sweet sorghum—.16, Sorghum—.35, Millet—.66, and P—.05.

There was a significant decrease in the number of crop‐raiding incidents from 2008 to 2013, with the number of raids dropping from 157.75 in 2008 to 6.87 in 2013, a slight increase between 2015 and 2018 (Appendix S1–S5).

Field RV's significantly varied depending on which crop was present on the farm (*F* = 16.8, *df* = 84, *p* < .0001) (Table 3). Pearson's correlation showed that an increase in the number of crop types grown in a field significantly reduced the vulnerability of that particular field from elephant raiding (*r* = −0.680, *df* = 1346, *p* = .00) (Appendix S1–S5). The location (villages) also influenced elephant raiding (*F* = 5.58, *df* = 12, *p* < .0001), with fields in Xakao, Beetsha, Mogotho, Ngarange, and Seronga having a higher risk of being raided (mean RV ≥3.50) compared with those in Eretsha, Tobera, Kauxwi, and Mohembo (mean RV = ≤2.96) (Table 3). There were significant differences in crop types damaged between villages (Appendix S1–S5).

**TABLE 3:** Mean RVs of fields in villages in the eastern Okavango Panhandle during the period of 2008–2018.

Villages	Number of fields (*n*)	Crops planted in fields	Mean risk of raiding for fields (RV)	Std error
Beetsha	155	Mi^(151)^, Ma^(131)^, So^(112)^, Wa^(115)^, Gr^(60)^, Pu^(99)^, Sw^(99)^, Co^(96)^	3.65	0.08
Eretsha	117	Mi^(117)^, Ma^(93)^, So^(64)^, Wa^(75)^, Gr^(26)^, Pu^(39)^, Sw^(38)^, Co^(43)^	2.96	0.09
Gudigwa	52	Mi^(52)^, Ma^(49)^, So^(39)^, Wa^(43)^, Gr^(18)^, Pu^(32)^, Sw^(18)^, Co^(28)^	3.43	0.10
Gunotsoga	134	Mi^(131)^, Ma^(113)^, So^(84)^, Wa^(100)^, Gr^(47)^, Pu^(69)^, Sw^(62)^, Co^(61)^	3.34	0.07
Kauxwi	81	Mi^(77)^, Ma^(40)^, So^(6)^, Wa^(36)^, Gr^(11)^, Pu^(12)^, Sw^(6)^, Co^(25)^	2.22	0.08
Mogotho	118	Mi^(114)^, Ma^(104)^, So^(85)^, Wa^(91)^, Gr^(59)^, Pu^(65)^, Sw^(65)^, Co^(68)^	3.62	0.07
Mohembo	101	Mi^(70)^, Ma^(34)^, So^(11)^, Wa^(16)^, Gr^(3)^, Pu^(4)^, Sw^(11)^, Co^(12)^	2.03	0.08
Mokgacha	27	Mi^(27)^, Ma^(23)^, So^(22)^, Wa^(17)^, Gr^(7)^, Pu^(5)^, Sw^(14)^, Co^(8)^	3.23	0.17
Ngarange	69	Mi^(65)^, Ma^(65)^, So^(61)^, Wa^(58)^, Gr^(29)^, Pu^(44)^, Sw^(40)^, Co^(28)^	3.60	0.10
Sekondomboro	142	Mi^(139)^, Ma^(132)^, So^(75)^, Wa^(118)^, Gr^(43)^, Pu^(47)^, Sw^(102)^, Co^(82)^	3.33	0.06
Seronga	156	Mi^(141)^, Ma^(144)^, So^(126)^, Wa^(110)^, Gr^(70)^, Pu^(90)^, Sw^(69)^, Co^(77)^	3.50	0.06
Tobera	134	Mi^(130)^, Ma^(109)^, So^(61)^, Wa^(43)^, Gr^(14)^, Pu^(28)^, Sw^(17)^, Co^(32)^	2.44	0.09
Xakao	61	Mi^(59)^, Ma^(58)^, So^(46)^, Wa^(50)^, Gr^(40)^, Pu^(34)^, Sw^(34)^, Co^(44)^	3.69	0.12

*Note*: The superscript numbers indicate the number of fields where the crop was available in the villages.

Abbreviations: Co, cowpea; Gr, groundnut; Mi, millet; Ma, maize; Pu, pumpkin; So, sorghum; Sw, sweet sorghum; Wa, watermelon.

## 4 DISCUSSION

Human–elephant conflict is complex, and there are many factors that influence crop‐raiding patterns by elephants (Hoare, 2012; Songhurst, 2017). Our study shows that crop type and crop diversity within a field are key factors that farmers should consider, despite their field locations, when trying to reduce the risk of elephant crop raiding. Cereal crops (millet, maize, and sorghum) faced a higher risk of crop raiding than leguminous plants (cowpea and groundnut). Likewise, our results showed a significant negative correlation between the number of crop types planted and the raiding vulnerability (RV) for the farm. The more crop types the field had, the lower the RV. Due to this variation in crop‐raiding risk, putting a high‐risk crop into the farm increased the potential of the farm getting raided and increased risks of crop loss. This association presents an opportunity for farmers to diversify their cropping strategy by planting lower risk crops and a wider diversity of crops to reduce the vulnerability to raiding and ultimately increase crop yields and food security in a human–elephant landscape.

Many farmers in the study area rarely grow a single crop, and there is always a likelihood that less susceptible crops are included in the field. This may explain the low RV found for all the fields. Although the increased diversity in the form of species richness is important in reducing RV, the characteristics of a crop species are more critical. For instance, in this study, a field with four crops (maize, millet, sorghum, and watermelon) had an RV of 2.73 and another field with four different crop groups (groundnut, cowpea, pumpkin, and sweet sorghum) had an RV of 1.80. These two crop diversities were significantly different in their susceptibility to elephant crop raiding despite both combinations having four crop types. The latter diversity had a lower chance of being raided, possibly because of the presence of groundnut and cowpea. When making crop combinations for planting, farmers should be advised to include crops with a lower IR to reduce the fields' risk value to elephant crop raiding (Nijman & Nekaris, 2010). The above findings further indicate that a functional group of a food crop is also a critical factor to consider when trying to reduce incidents of crop raiding by elephants. In this study, we found that elephants were averse to certain crops, and the aversion increased when more crops with repulsive characteristics from other functional groups were added to the mixture of crops in the field. As a result, a combination of the crops from different functional groups showed varying susceptibility to elephant crop raiding.

Determining crop susceptibility to elephant crop raiding adds another promising dimension to mitigation efforts against human–elephant conflict. These crop diversification measures would need to complement other mitigation measures such as fences, flashlights, drums, and chili pepper, in order to reinforce the guarding (Montgomery et al., 2022; Sitati et al., 2005). Farmers invest more resources such as time and mechanical equipment to guard against elephant crop raiding (Bond, 2015), and the supply of modern mitigation tools is often a limiting factor to subsistence farmers in the eastern Okavango Panhandle (Noga et al., 2015). However, the use of crops with low susceptibility to elephant damage together with other noninvasive and locally available mitigation measures can be a sustainable solution to the high human–elephant conflict in the eastern Okavango Panhandle and other regions with similar issues.

A significant decrease in the number of elephant crop‐raiding incidents in the eastern Okavango Panhandle was observed over the past decade. Certain crops, individually or in combination, deterred crop raiding, and susceptibility of crops to raiding varied between crop types and functional groups. The findings support Hoare's (2012) and Nyirenda et al.'s (2018) observations that some crop types are more susceptible to elephant damage than others. Most of the crops, which elephants raided in this study, are also preferred human food crops (Marumo et al., 2014), which farmers depend on for food and financial income. In earlier studies in the eastern Okavango Panhandle, elephants were found to strongly favor millet, which is a principal food in the area (Songhurst, 2017). Our findings similarly demonstrated the preference and risk brought about by growing millet. In Ghana, elephants were observed to raid cereal crops such as maize and sorghum more frequently than other crops (Monney et al., 2010). Similar findings were reported elsewhere (Barua et al., 2013; Das et al., 2014; Goswami et al., 2015). Preference by elephants for cereal crops that farmers are dependent on for food and economic progress presents a challenging situation for reducing human–elephant conflict in Africa. Competition for food aggravates the conflict and leads to a reduced food supply from the farms (Sitati et al., 2005), loss of surplus harvest and potential income (Gontse et al., 2018).

The melon category comprising pumpkins and watermelons was the second most preferred and raided group. The high‐water content of the melon crops renders them an excellent alternative source of water, especially during the dry season (Warner, 2008). On the contrary, legumes consisting of groundnut and cowpea were the least preferred and the least susceptible to elephant raiding. These findings are consistent with Mingyong (2008), who found that beans, which were leguminous crops as well were more resilient to elephant crop raiding than maize. Similarly, the Asian elephant (*Elephas maximus*) in Cambodia and African savanna elephant (*Loxodonta africana*) in Tanzania were not interested in groundnut and beans, respectively (Kiffner et al., 2021; Webber et al., 2011). A negative correlation between crop raiding by African savanna elephants and damage on beans was also recorded in Burkina Faso in West Africa, where cereal crops (sorghum, maize, and millet) were similarly most preferred by elephants compared with beans (Compaore et al., 2020). As already discussed in previous studies, the differential preference of a particular crop over the other by elephants possibly emanates from the ease of access to the crop, caloric or nutritional content, and palatability (Monney et al., 2010; Songhurst et al., 2015; Vogel et al., 2020). The decline in elephant raiding incidents observed in this study between 2008 and 2013 coincided with the period when rainfall was lower and many farmers had planted cowpea (Statistics Botswana, 2016, 2019), which is a crop that elephants are averse to. During this period, there was an increase from 939 to 2021 individual farmers who planted cowpea instead of cereal crops (Statistics Botswana, 2019). Similarly, in 2015, fewer farmers (172) planted cowpea than the 2021 farmers in 2013 (Statistics Botswana, 2019), potentially resulting in an increase in crop‐raiding incidents. Adopting less vulnerable crops to elephant raiding, such as groundnut and cowpea, can be an effective and sustainable strategy in mitigating human–elephant conflict in agro‐ecological systems. Notwithstanding that, there is still a need to determine why some leguminous crops such as pigeon peas were highly damaged by the elephants than other crops in countries such as Tanzania (Snyder et al., 2021). We acknowledge that elephants in a similar way like other animals can select resources based on their relative abundance and availability and that they can tolerate even plants rich in toxic substances (chemical defenses/antifeedants). Also, the growth stage of the plant can dictate its repulsiveness, for example, elephants can feed on chili plants before the fruits ripen, but once mature and ripe, the chili plant becomes repulsive and can be an effective buffer crop (Matsika et al., 2020).

In the eastern Okavango Panhandle, farmers still prefer some crops despite these crops also being preferred by elephants. The melons are second in terms of preference by farmers and elephants. The preference of these crops by farmers increases opportunity costs for farmers since the government of the Republic of Botswana does not compensate for melons when damaged by wildlife, including elephants (Department of Wildlife and National Parks, 2013). For the crops assessed in this study, the Botswana government only compensates for wildlife damages on maize, sorghum, millet, cowpea, and groundnut (Department of Wildlife and National Parks, 2013). Farmers, therefore, see the exclusion of some crops from the compensation scheme as unjustifiable. Moreover, it often leads many farmers failing to report crop‐raiding incidents and crop losses as reporting does not make any financial difference to them (DeMotts & Hoon, 2012). Issues underlying attachment to certain crops such as cultural attachment, popularity, economic reasons, ease of getting seeds (Guei et al., 2011) often make farmers to continue planting highly susceptible crops despite being aware of the unrecoverable losses in case of elephant raids.

Our results suggest that the susceptibility of the fields to elephant raids could be minimized by carefully selecting crop types and combinations not susceptible to elephant damage, and this will enhance food security for the local farmers. We recommend that human–elephant coexistence strategies have a strong focus on educating farmers to select and grow combinations of low‐risk crops. An effective crop diversification strategy will include: different types of crops from different functional groups; less susceptible crops to elephant raiding such as legumes; and less palatable crops to elephants. Further research is needed to experimentally evaluate the effects of planting different combinations of food crops and other crop types as a strategy to minimize the risk of crop raiding by elephants.

## AUTHOR CONTRIBUTIONS

**Tiroyaone A. Matsika:** Conceptualization (equal); formal analysis (lead); investigation (equal); project administration (equal); writing – original draft (lead); writing – review and editing (lead). **Gaseitsiwe S. Masunga:** Investigation (supporting); writing – original draft (supporting); writing – review and editing (supporting). **Anastacia Makati:** Formal analysis (supporting); investigation (equal); writing – original draft (supporting). **Graham McCulloch:** Funding acquisition (supporting); project administration (equal); writing – review and editing (equal). **Anna Songhurst:** Funding acquisition (lead); methodology (equal); supervision (equal); writing – review and editing (equal). **Amanda Stronza:** Funding acquisition (equal); supervision (equal). **Joseph Adjetey:** Project administration (equal); supervision (lead); writing – original draft (supporting); writing – review and editing (equal). **Motshwari Obopile:** Methodology (equal); project administration (supporting); supervision (equal).

## CONFLICT OF INTEREST STATEMENT

The authors of this work declare that there is no conflict of interest to declare.

## IMPACT STATEMENT

Susceptibility of crops to elephant raiding varies amongst crop types and functional groups in northern Botswana.

## Supporting information


Appendix S1.

## Data Availability

Data are stored at Botswana University of Agriculture and Natural Resources Library, Gaborone, Botswana and Ecoexist Trust offices in Maun, and deposited in the Mendeley repository. https://data.mendeley.com/datasets/jfjx8752c7/1, DOI:10.17632/jfjx8752c7.1

## References

[ece39910-bib-0001] Barua, M. , Bhagwat, S. A. , & Jadhav, S. (2013). The hidden dimensions of human–wildlife conflict: Health impacts, opportunity and transaction costs. Biological Conservation, 157, 309–316.

[ece39910-bib-0002] Bond, J. (2015). Making sense of human‐elephant conflict in Laikipia County, Kenya. Society & Natural Resources, 28(3), 312–327.

[ece39910-bib-0003] Buchholtz, E. K. , Redmore, L. , Fitzgerald, L. A. , Stronza, A. , Songhurst, A. , & McCulloch, G. (2019). Temporal partitioning and overlapping use of a shared natural resource by people and elephants. Frontiers in Ecology and Evolution, 7, 117.

[ece39910-bib-0004] Central Statistics Office, Botswana . (2011). Human population and housing census. Government of Botswana. Available from:https://www.statsbots.org.bw/sites/default/files/publications/Population%20and%20Housing%20Census%202011%20%20Dissemination%20seminar%20report.pdf

[ece39910-bib-0005] Chang, A. , de Souza, N. , Muya, J. , Keyyu, J. , Mwakatobe, A. , Malugu, L. , Ndossi, H. P. , Konuche, J. , Omondi, R. , Mpinge, A. , Hahn, N. , Palminteri, S. , & Olson, D. (2016). Scaling‐up the use of chili fences for reducing human‐elephant conflict across landscapes in Tanzania. Tropical Conservation Science, 9, 921–930.

[ece39910-bib-0006] Chase, M. J. , Schlossberg, S. , Griffin, C. R. , Bouché, P. J. C. , Djene, S. W. , Elkan, P. W. , Ferreira, S. , Grossman, F. , Kohi, E. M. , Landen, K. , Omondi, P. , Peltier, A. , Selier, S. A. J. , & Sutcliffe, R. (2016). Continent‐wide survey reveals massive decline in African savannah elephants. Peer Journal, 4, 1–24. 10.7717/peerj.2354 PMC5012305 27635327

[ece39910-bib-0007] Compaore, A. , Sirima, D. , Hema, E. M. , Doamba, B. , Ajong, S. N. , Di Vittorio, M. , & Luiselli, L. (2020). Correlation between increased human‐elephant conflict and poaching of elephants in Burkina Faso (West Africa). European Journal of Wildlife Research, 66(1), 1–9.

[ece39910-bib-0008] Das, B. J. , Saikia, B. N. , Baruah, K. K. , Bora, A. , & Bora, M. (2014). Nutritional evaluation of fodder, its preference and crop raiding by wild Asian elephant (*Elephas maximus*) in Sonitpur District of Assam, India. Veterinary World, 7, 1082–1089.

[ece39910-bib-0009] DeMotts, R. , & Hoon, P. (2012). Whose elephants? Conserving, compensating, and competing in northern Botswana. Society and Natural Resources, 25, 837–851.

[ece39910-bib-0010] Department of Meteorological Services, Botswana . (2016). Seasonal rainfall outlook for October to December (OND) 2016, Novermber to January (NDJ) 2016–17, December to February (DJF) 2016–17 and January to March (JFM) 2017. Available from: FILE:///H:/HECPHD/REVIEWS/METREOLOGY.PDF

[ece39910-bib-0011] Department of Wildlife and National Parks . (2013). Compensation guidelines for damages caused by elephants and lion. In Ministry of Environment, Wildlife and Tourism. Government of Botswana.

[ece39910-bib-0012] Gontse, K. , Mbaiwa, J. E. , & Thakadu, O. T. (2018). Effects of wildlife crop raiding on the livelihoods of arable farmers in Khumaga, Boteti sub‐district, Botswana. Development Southern Africa, 35, 791–802.

[ece39910-bib-0013] Goswami, V. R. , Medhi, K. , Nichols, J. D. , & Oli, M. K. (2015). Mechanistic understanding of human–wildlife conflict through a novel application of dynamic occupancy models. Conservation Biology, 29, 1100–1110. 25757801 10.1111/cobi.12475

[ece39910-bib-0014] Gross, E. M. , McRobb, R. , & Gross, J. (2016). Cultivating alternative crops reduces crop losses due to African elephants. Journal of Pest Science, 89(2), 497–506.

[ece39910-bib-0015] Guei, R. G. , Barra, A. , & Silué, D. (2011). Promoting smallholder seed enterprises: Quality seed production of rice, maize, sorghum and millet in northern Cameroon. International Journal of Agricultural Sustainability, 9, 91–99.

[ece39910-bib-0016] Guerbois, C. , Chapanda, E. , & Fritz, H. (2012). Combining multi‐scale socio‐ecological approaches to understand the susceptibility of subsistence farmers to elephant crop raiding on the edge of a protected area. Journal of Applied Ecology, 49, 1149–1158.

[ece39910-bib-0017] Hauke, J. , & Kossowski, T. (2011). Comparison of values of Pearson's and Spearman's correlation coefficients on the same set of data. Quaestiones Geographicae, 30, 87–93.

[ece39910-bib-0018] Hoare, R. (2012). Lessons from 15 years of human‐elephant conflicts mitigation: Management considerations involving biological, physical and governance issues in Africa. Pachyderm, 51, 60–74.

[ece39910-bib-0019] Hoare, R. E. (1999). Data collection and analysis protocol for human‐elephant conflict situations in Africa. UICN African Elephant Specialist Group Human‐Elephant Conflict Working Group.

[ece39910-bib-0020] Jackson, T. P. , Mosojane, S. , Ferreira, S. M. , & van Aarde, R. J. (2008). Solutions for elephant Loxodonta africana crop raiding in northern Botswana: Moving away from symptomatic approaches. Oryx, 42, 83–91.

[ece39910-bib-0021] Kansky, R. , & Knight, A. T. (2014). Key factors driving attitudes towards large mammals in conflict with humans. Biological Conservation, 179, 93–105.

[ece39910-bib-0022] Kiffner, C. , Schaal, I. , Cass, L. , Peirce, K. , Sussman, O. , Grueser, A. , & Kioko, J. (2021). Perceptions and realities of elephant crop raiding and mitigation methods. Conservation Science and Practice, 3(3), e372.

[ece39910-bib-0023] King, B. , Shinn, J. , Crews, K. , & Young, K. (2016). Fluid waters and rigid livelihoods in the Okavango Delta of Botswana. Landscape, 5, 16.

[ece39910-bib-0024] Marumo, D. S. , Tselaesele, N. M. , Batlang, U. , Nthoiwa, G. , & Jansen, R. (2014). Poverty and social impact analysis of the integrated support Programme for arable agriculture development in Botswana. UNDP‐UNEP‐GoB PovertyEnvironment Initiative (PEI) Working Paper no. 2. Gaborone, Botswana.

[ece39910-bib-0025] Matsika, T. A. , Adjetay, J. A. , Obopile, M. , Songhurst, A. C. , McCulloch, G. , & Stronza, A. (2020). Alternative crops as a mitigation measure for elephant crop raiding in the eastern Okavango panhandle. Pachyderm, 61, 140–152.

[ece39910-bib-0026] Mayberry, A. L. , Hovorka, A. J. , & Evans, K. E. (2017). Well‐being impacts of human‐elephant conflict in Khumaga, Botswana: Exploring visible and hidden dimensions. Conservation and Society, 15, 280–291.

[ece39910-bib-0027] Mingyong, C. A. (2008). Study on ecological mechanisms of man‐elephant conflicts in Xishuangbanna biosphere reserve, Dissertation. Institute of Xishuangbanna National Nature Reserve, Jinghong, Yunnan, China.

[ece39910-bib-0028] Monney, K. A. , Dakwa, K. B. , & Wiafe, E. D. (2010). Assessment of crop raiding situation by elephants (Loxodonta africana cyclotis) in farms around Kakum conservation area, Ghana. International Journal of Biodiversity and Conservation, 2, 243–249.

[ece39910-bib-0029] Montgomery, R. A. , Raupp, J. , Mukhwana, M. , Greenleaf, A. , Mudumba, T. , & Muruthi, P. (2022). The efficacy of interventions to protect crops from raiding elephants. Ambio, 51, 716–727. 34173175 10.1007/s13280-021-01587-x PMC8800974

[ece39910-bib-0030] Mosojane, S. (2004). Human‐elephant conflict along the Okavango panhandle in northern Botswana, research report submitted in partial fulfilment of the requirements for a degree of MSc (conservation ecology and planning). Department of Zoology and Entomology Faculty of Agriculture and Natural Sciences, University of Pretoria, South Africa.

[ece39910-bib-0031] Motsholapheko, M. R. , Kgathi, D. L. , & Vanderpos, C. (2012). Rural livelihood diversification: A household adaptive strategy against flood variability in the Okavango Delta, Botswana. Agrekon, 51, 41–62.

[ece39910-bib-0032] Naughton, L. , Rose, R. , & Treves, A. (1998). Predicting patterns of crop damage by wildlife around Kibale National Park, Uganda. Conservation Biology, 12, 156–168.

[ece39910-bib-0033] Nijman, V. , & Nekaris, K. A. I. (2010). Testing a model for predicting primate crop‐raiding using crop‐ and farm‐specific risk values. Applied Animal Behaviour Science, 127, 125–129.

[ece39910-bib-0034] Noga, S. R. , Kolawole, O. D. , Thakadu, O. , & Masunga, G. (2015). Small farmers' adoption behaviour: Uptake of elephant crop‐raiding deterrent innovations in the Okavango Delta, Botswana. African Journal of Science, Technology, Innovation and Development, 7, 408–419.

[ece39910-bib-0035] Nyirenda, V. R. , Nkhata, B. A. , Tembo, O. , & Siamundele, S. (2018). Elephant crop damage: Subsistence farmers' social vulnerability, livelihood sustainability and elephant conservation. Sustainability, 10, 3572.

[ece39910-bib-0036] Osborn, F. V. (2002). Capsicum oleoresin as an elephant repellent: Field trials in the communal lands of Zimbabwe. Journal of Wildlife Management, 66, 674–677. 10.2307/3803133

[ece39910-bib-0037] Owen‐Smith, N. , & Chafota, J. (2012). Selective feeding by a megaherbivore, the African elephant (Loxodonta africana). Journal of Mammalogy, 93(3), 698–705.

[ece39910-bib-0038] Parker, G. E. , & Osborn, F. V. (2006). Investigating the potential for chilli capsicum spp. to reduce human‐wildlife conflict in Zimbabwe. Oryx, 40, 343–346.

[ece39910-bib-0039] Pozo, R. A. , Coulson, T. , McCulloch, G. , Stronza, A. L. , & Songhurst, A. C. (2017). Determining baselines for human‐elephant conflict: A matter of time. PLoS One, 12(6), e0178840. 28582425 10.1371/journal.pone.0178840 PMC5459443

[ece39910-bib-0040] Priston, N. E. C. , & Underdown, S. J. (2009). A simple method for calculating the likelihood of crop damage by primates: An epidemiological approach. International Journal of Pest Management, 55, 51–56.

[ece39910-bib-0041] R Core Team . (2013). R: A language and environment for statistical computing. R Foundation for Statistical Computing. http://www.R‐project.org/

[ece39910-bib-0042] Regmi, G. R. , Nekaris, K. A. I. , Kandel, K. , & Nijman, V. (2013). Crop‐raiding macaques: Predictions, patterns and perceptions from Langtang National Park. Nepal. Endangered Species Research, 20, 217–226.

[ece39910-bib-0043] Sitati, N. W. , Walpole, M. J. , & Leader‐williams, N. (2005). Factors affecting susceptibility of farms to crop raiding by African elephants: Using a predictive model to mitigate. Journal of Applied Ecology, 42, 1175–1182.

[ece39910-bib-0044] Snyder, K. D. , Mneney, P. , Benjamin, B. , Mkilindi, P. , & Mbise, N. (2021). Seasonal and spatial vulnerability to agricultural damage by elephants in the western Serengeti, Tanzania. Oryx, 55(1), 139–149.

[ece39910-bib-0045] Songhurst, A. (2017). Measuring human–wildlife conflicts: Comparing insights from different monitoring approaches. Wildlife Society Bulletin, 41, 351–361.

[ece39910-bib-0046] Songhurst, A. , Chase, M. , & Coulson, T. (2015). Using simulations of past and present elephant (*Loxodonta africana*) population numbers in the Okavango Delta panhandle, Botswana to improve future population estimates. Wetlands Ecology and Management, 23, 583–602.

[ece39910-bib-0047] Songhurst, A. , & Coulson, T. (2014). Exploring the effects of spatial autocorrelation when identifying key drivers of wildlife crop‐raiding. Ecology and Evolution, 4, 582–593. 25035800 10.1002/ece3.837 PMC4098139

[ece39910-bib-0048] Songhurst, A. , McCulloch, G. , & Coulson, T. (2016). Finding pathways to human–elephant coexistence: A risky business. Oryx, 50, 713–720.

[ece39910-bib-0049] Statistics Botswana . (2016). Botswana environment statistics 2016 revised version (RV). Statistics Botswana, Gaborone. https://www.statsbots.org.bw/sites/default/files/publications/Botswana%20Environment%20Statistics%20Report%20Rv.pdf

[ece39910-bib-0050] Statistics Botswana . (2019). Botswana environment statistics: Water and climate digest. Statistics Botswana, Gaborone. http://www.statsbots.org.bw/sites/default/files/publications/Environment%20Statistic%20Water%20%26%20Climate%20Digest%202019.pdf

[ece39910-bib-0051] Thouless, C. R. , Dublin, J. J. , Blanc, D. P. , Skinner, T. E. , Daniel, R. D. , Taylor, F. , Maisels, H. L. , & Bouché, P. (2016). African elephant status report 2016. Occasional paper series of the IUCN species survival commission, 60.

[ece39910-bib-0052] Tiller, L. N. , Humle, T. , Amin, R. , Deere, N. J. , Lago, B. O. , Leader‐Williams, N. , Sinoni, F. K. , Sitati, N. , Walpole, M. , & Smith, R. J. (2021). Changing seasonal, temporal and spatial crop‐raiding trends over 15 years in a human‐elephant conflict hotspot. Biological Conservation, 254, 108941.

[ece39910-bib-0053] Vogel, S. M. , Blumenthal, S. A. , de Boer, W. F. , Masake, M. , Newton, I. , Songhurst, A. C. , McCulloch, G. , Stronza, A. , Henley, M. D. , & Coulson, T. (2020). Timing of dietary switching by savannah elephants in relation to crop consumption. Biological Conservation, 249, 108703.

[ece39910-bib-0054] Warner, M. Z. (2008). Examining human‐elephant conflict in southern Africa: Causes and options for coexistence (Master thesis) (p. 101). University of Pennsylvania, United States of America. http://repository.upenn.edu/cgi/viewcontent.cgi?article=1021&context=mes_capstone

[ece39910-bib-0055] Webber, C. E. , Sereivathana, T. , Maltby, M. P. , & Lee, P. C. (2011). Elephant crop‐raiding and human‐elephant conflict in Cambodia: Crop selection and seasonal timings of raids. Flora and Fauna International, 45, 243–251.

