# Supplementary material for: Crop diversity and susceptibility of crop fields to elephant raids in eastern Okavango Panhandle, northern Botswana
Source: Ecol Evol. 2023 Mar 21;13(3):e9910. doi: 10.1002/ece3.9910 (PMC10030231; doi:10.1002/ece3.9910)
Supplement: Supplementary file 1 — Appendix S1. [file ECE3-13-e9910-s001.docx]

**Supporting Information**

**Appendix S1** Crop raiding over the years

| Source | DF | SS | MS | F | P |
| --- | --- | --- | --- | --- | --- |
| Crops | 7 | 51495 | 7356.4 | 9.16 | 0.000 |
| Year | 9 | 139063 | 15451.5 | 19.23 | 0.000 |
| Error | 63 | 50613 | 803.4 |  |  |
| Total | 79 | 241171 |  |  |  |
|  |  | S = 28.34 | R-Sq = 79.01% | R-Sq(adj) = 73.68% |  |

**. Significant at the 0.05 level.

**Appendix S2** Influence of crop diversity on fields’ vulnerability to crop raiding

|  | | Number of crops Planted | Field RV |
| --- | --- | --- | --- |
| Number of crops Planted | Pearson Correlation | 1 | -.680^**^ |
|  | Sig. (2-tailed) |  | .000 |
|  | N | 1347 | 1346 |
| Field RV | Pearson Correlation | -.680^**^ | 1 |
|  | Sig. (2-tailed) | .000 |  |
|  | N | 1346 | 1346 |
| **. Correlation is significant at the 0.01 level (2-tailed). | | | |

**Appendix S3** Correlation procedure between crop available, crop damaged, and IR

| Pearson Correlation Coefficients Prob > \|r\| under H0: Rho=0 Number of Observations | | | | | |
| --- | --- | --- | --- | --- | --- |
|  | Crop available | | Crop damaged | | IR |
| Crop available | | 1.00000  819 | | 0.72769 <.0001 812 | 0.01696 0.6338 792 |
| Crop damaged | | 0.72769 <.0001 812 | | 1.00000  812 | 0.38711 <.0001 792 |
| IR | | 0.01696 0.6338 792 | | 0.38711 <.0001 792 | 1.00000  793 |

**Appendix S4** The number of elephant crop raiding incidents in different villages in the eastern Okavango Panhandle in 2008 (a) and 2018 (b). The sizes of the green-filled circles indicate the differences in the number of crop raiding incidents in different villages within one particular year.


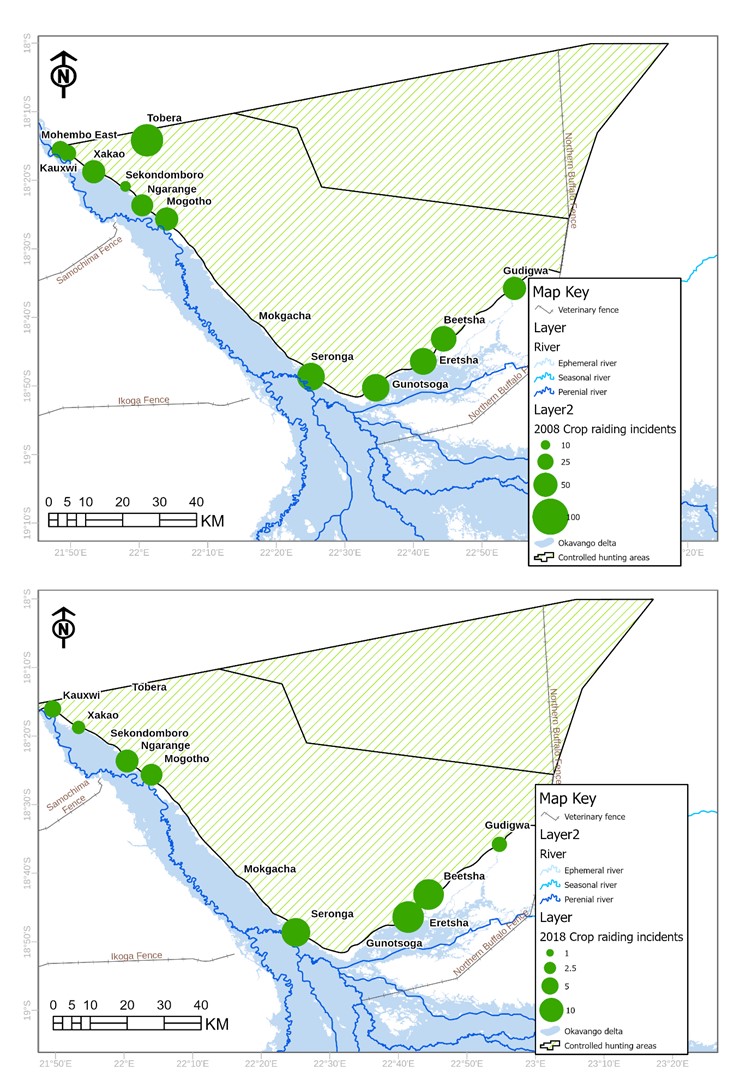


**Appendix S5** The GLM procedure, dependent variable: crop damaged

| Source | DF | Sum of Squares | Mean Square | F Value | Pr > F |
| --- | --- | --- | --- | --- | --- |
| Model | 103 | 11371.38903 | 110.40184 | 2.32 | <.0001 |
| Error | 708 | 33626.84127 | 47.49554 |  |  |
| Corrected Total | 811 | 44998.23030 |  |  |  |

| R-Square | Coeff Var | Root MSE | Crop damaged Mean |
| --- | --- | --- | --- |
| 0.252707 | 138.6880 | 6.891701 | 4.969212 |

| Source | DF | Type III SS | Mean Square | F Value | Pr > F |
| --- | --- | --- | --- | --- | --- |
| Village | 12 | 3177.952178 | 264.829348 | 5.58 | <.0001 |
| Crop | 7 | 5593.495462 | 799.070780 | 16.82 | <.0001 |
| Village*Crop | 84 | 2210.155761 | 26.311378 | 0.55 | 0.9995 |

**Appendix S6** The GLM procedure, t Test (LSD) for crop damaged in different villages

| Village Comparison @ critical value of t=1.96 | Difference Between Means | 95% Confidence Limits | | P level |
| --- | --- | --- | --- | --- |
| Seronga - Mogotho | 2.454 | 0.12 | 4.789 | *** |
| Seronga - Gunotsoga | 2.81 | 0.409 | 5.212 | *** |
| Seronga - Tobera | 2.885 | 0.551 | 5.219 | *** |
| Seronga - Sekondomboro | 3.222 | 0.811 | 5.633 | *** |
| Seronga - Mohembo | 5.104 | 2.746 | 7.461 | *** |
| Seronga - Eretsha | 5.123 | 2.721 | 7.524 | *** |
| Seronga - Ngarange | 5.873 | 3.471 | 8.274 | *** |
| Seronga - Gudigwa | 6.248 | 3.846 | 8.649 | *** |
| Seronga - Kauxwi | 6.457 | 4.065 | 8.849 | *** |
| Seronga - Mokgacha | 7.17 | 4.232 | 10.107 | *** |
| Beetsha - Mohembo | 2.731 | 0.383 | 5.079 | *** |
| Beetsha - Eretsha | 2.75 | 0.358 | 5.142 | *** |
| Beetsha - Ngarange | 3.5 | 1.108 | 5.892 | *** |
| Beetsha - Gudigwa | 3.875 | 1.483 | 6.267 | *** |
| Beetsha - Kauxwi | 4.084 | 1.702 | 6.467 | *** |
| Beetsha - Mokgacha | 4.797 | 1.867 | 7.726 | *** |
| Xakao - Mohembo | 2.729 | 0.295 | 5.162 | *** |
| Xakao - Eretsha | 2.748 | 0.272 | 5.224 | *** |
| Xakao - Ngarange | 3.498 | 1.022 | 5.974 | *** |
| Xakao - Gudigwa | 3.873 | 1.397 | 6.349 | *** |
| Xakao - Kauxwi | 4.082 | 1.615 | 6.549 | *** |
| Xakao - Mokgacha | 4.795 | 1.796 | 7.793 | *** |
| Mogotho - Mohembo | 2.649 | 0.37 | 4.929 | *** |
| Mogotho - Eretsha | 2.668 | 0.344 | 4.993 | *** |
| Mogotho - Ngarange | 3.418 | 1.094 | 5.743 | *** |
| Mogotho - Gudigwa | 3.793 | 1.469 | 6.118 | *** |
| Mogotho - Kauxwi | 4.003 | 1.688 | 6.318 | *** |
| Mogotho - Mokgacha | 4.715 | 1.841 | 7.59 | *** |
| Gunotsoga - Ngarange | 3.063 | 0.671 | 5.454 | *** |
| Gunotsoga - Gudigwa | 3.438 | 1.046 | 5.829 | *** |
| Gunotsoga - Kauxwi | 3.647 | 1.264 | 6.03 | *** |
| Gunotsoga - Mokgacha | 4.359 | 1.43 | 7.289 | *** |
| Tobera - Ngarange | 2.988 | 0.663 | 5.312 | *** |
| Tobera - Gudigwa | 3.363 | 1.038 | 5.687 | *** |
| Tobera - Kauxwi | 3.572 | 1.257 | 5.887 | *** |
| Tobera - Mokgacha | 4.285 | 1.41 | 7.159 | *** |
| Sekondomboro - Ngarange | 2.651 | 0.249 | 5.052 | *** |
| Sekondomboro - Gudigwa | 3.026 | 0.624 | 5.427 | *** |
| Sekondomboro - Kauxwi | 3.235 | 0.843 | 5.627 | *** |
| Sekondomboro - Mokgacha | 3.947 | 1.01 | 6.885 | *** |

Variation of elephant raiding in above villages significant at P=0.05***, any comparison not presented was not statistically significant.
